# Teleoncology Orientation of Low-Income Breast Cancer Patients during the COVID-19 Pandemic: Feasibility and Patient Satisfaction

**DOI:** 10.1055/s-0041-1739425

**Published:** 2021-12-06

**Authors:** Roberta Amparado Miziara, Jonathan Yugo Maesaka, Danielle Ramos Martin Matsumoto, Laura Penteado, Ariane Andrade dos Santos Anacleto, Tarso Augusto Duenhas Accorsi, Karine De Amicis Lima, Eduardo Cordioli, Gabriel Salum D´Alessandro

**Affiliations:** 1 Breast Surgery Department, Hospital Vila Santa Catarina, São Paulo, SP, Brazil; 2 Telemedicine Department, Hospital Israelita Albert Einstein, São Paulo, SP, Brazil

**Keywords:** telemedicine, breast cancer, pandemics, patient satisfaction, telemedicina, câncer de mama, pandemia, satisfação do patience

## Abstract

**Objective** The present study aims to assess the feasibility and patient satisfaction of teleoncology orientation in a vulnerable population of breast cancer patients assessed in a government health system during the coronavirus pandemic in 2020.

**Methods** Eligible patients received an invitation to receive remote care to minimize exposure to an environment in which the risk of respiratory infection was present. The means of communication was telephone through an application that allows free conversation with no charge. An anonymous-response questionnaire based on a Likert-type scale was sent through a cell phone application or e-mail directly to each patient or close relative of the patient immediately after teleconsultation. Responses to the questions, which addressed utility, facility, interface quality, interaction quality, reliability, satisfaction, and interest in future evaluation, were compiled and analyzed.

**Results** A total of 176 eligible patients scheduled for consultation were evaluated and 98 were included. Seventy (71.4%) successfully undertook the teleorientation. The questionnaire was submitted by 43 (61.4%) patients. The overall teleoncology orientation was classified as very positive by 41 (95.3%) patients. Specifically, regarding the questionnaire items, 43 (100%) patients scored 4 or 5 (agreed that the teleconsultation was beneficial) concerning the facility, followed by 42 (97.2%) for the interface quality, 41 (95.3%) for both utility and interaction quality, 40 (93%) for satisfaction and interest in future evaluation, and, finally, 39 (90.6%) for reliability.

**Conclusion** Teleoncology orientation of low-income breast cancer patients is most feasible and leads to high patient satisfaction.

## Resumo

**Objetivo** O presente estudo teve como objetivo avaliar a viabilidade e satisfação em relação à orientação teleoncológica realizada em população vulnerável de pacientes com câncer de mama e provenientes do sistema público de saúde durante a pandemia do coronavírus em 2020.

**Métodos** Pacientes elegíveis foram agendados para atendimento remoto visando minimizar exposição a ambientes com risco de infecção respiratória. O meio de comunicação foi o telefone, pois permite conversa sem custos. Um questionário anônimo com base na escala Likert foi enviado através de aplicativo de telefone celular ou e-mail para paciente ou familiares, logo após a teleconsulta. As respostas, que abordavam utilidade, facilidade, qualidade da interface, qualidade da interação, confiabilidade, satisfação e interesse em avaliações futuras, foram compiladas e analisadas.

**Resultados** Um total de 176 pacientes elegíveis para teleconsulta foram avaliados e 98 foram incluídos. Setenta (71,4%) realizaram a teleorientação com sucesso. O questionário foi respondido por 43 (61,4%) pacientes. De maneira geral, a teleorientação foi classificada como muito positiva por 41 (95,3%) pacientes. Em relação aos itens avaliados, 43 (100%) pacientes pontuaram 4 ou 5 (concordaram que a teleconsulta era benéfica) em relação à facilidade do serviço, seguido por 42 (97,2%) para a qualidade da interface, 41 (95,3%) tanto para a utilidade quanto para a qualidade da interação, 40 (93%) para satisfação e interesse em avaliação futura e 39 (90,6%) para confiabilidade em relação ao método.

**Conclusão** A orientação teleoncológica em pacientes de baixa renda e com câncer de mama mostrou ser viável e com altas taxas de satisfação.

## Introduction

Telemedicine has become a key factor in the restructuring of the health system required by the COVID-19 pandemic for quick, economical, and safe medical assessment. 1 Especially to facilitate the public health system populations to access specialists for evaluation, telemedicine is an efficient method. 2 This method implies the potential to reduce the burden of high-morbidity chronic conditions. 3 Active cancer patients are at risk both for loss of medical follow-up and for severe COVID-19 infection presentation. 4

Breast cancer is very much prevalent but potentially curable. Its undertreatment has become a major focus of management and can be circumvented with frequent monitoring. 5 Teleoncology for breast cancer patients brings several benefits, such as telegenetics, remote chemotherapy supervision, symptom management, and palliative care. 6 However, the implementation of telemedicine faces several challenges among populations that lack education and economic resources. 7 Despite being conceptually advantageous, few studies have analyzed the role of telemedicine in treating cancer patients of the low-income group and, currently, the focus is on resolving issues inherent to the disease and not on guidance and satisfaction. 8 The present study aims to assess the feasibility and patient satisfaction of teleoncology orientation in a low-income population of breast cancer patients assessed in a public health system during the COVID-19 pandemic.

## Methods

### Population

The present study was approved by the local institutional review board. All enrolled patients provided written informed consent before inclusion. Between April and July 2020, during the COVID-19 pandemic, we prospectively enrolled all consecutive adult patients (≥ 18 years old) regularly scheduled for presential consultation at the breast cancer clinic of the Hospital Municipal Vila Santa Catarina, São Paulo, state of São Paulo, Brazil. The inclusion criteria for being offered teleoncology orientation were stable breast cancer, being on regular treatment, and a good presumed prognosis based on the last consultation registered with complete data in the institutional medical record. All patients were followed according to specific oncological institutional guidelines. Patients on surgical programs, bad clinical status, being prepared for new drug treatment, or waiting for a new oncologic diagnosis were excluded.

### Methodologies

Eligible patients received an invitation to receive remote care to minimize exposure to an environment in which the risk of respiratory infection was present. The selected means of communication for telemedicine was telephone through an application that allows free conversation with no charge or by landline if that was the only available means. The call was made at the same time as the previously scheduled face-to-face consultation and by the same surgeon who had on-time access to patient records. Telephone consultation took place as long as necessary according to clinical judgment. The teleconsultation was performed in synchronous interaction, with questions being asked and answered in real time, similar to face-to-face consultation. Clinical data and the epidemiological and socioeconomic profiles of the consulted patients were included from medical records. An anonymous-response questionnaire translated from English to Portuguese based on a Likert-type scale was sent through a cell phone application or e-mail directly to each patient or close relative of the patient immediately after teleconsultation. 9 Seven selected aspects (usefulness, easiness, interface quality, interaction quality, reliability, satisfaction, and interest in future use) were also compiled ( Table 1 ) and analyzed. The Likert-type scale is a psychometric questionnaire tool based on a sentence followed by a level of agreement scored on a scale ranging from (1) completely disagree to (5) completely agree. Since this type of questionnaire translated and validated into Portuguese is not available in Brazil, it was designed and translated based on the review article by Parmanto et al. 9

**Table 1:** Likert scale-adapted questionnaire

	1a - Targeting question
1	My problem was satisfactorily solved via phone guidance
2	I liked the phone guidance, as I did not have to go to the hospital to get my problem solved
3	Scheduling and telephone consultation were carried out correctly
4	Telephone consultation was pleasant
5	Telephone consultation was able to perform everything I had imagined
6	The phone call was a good method to talk to the doctor
7	I was able to tell the doctor everything I wanted to tell during the phone call
8	Telephone consultation was similar to a face-to-face consultation with the doctor
9	I felt safe about the management performed by the doctor through the phone call
10	I felt comfortable consulting with the doctor through a phone conversation
11	Telephone guidance is an acceptable way to talk to the doctor
12	I would again accept phone orientation
	**1b - Standard answer for all questions (Likert scale)**
	I completely agree
	I partially agree
	I neither agree nor disagree
	I partially disagree
	I completely disagree

### Statistical Analysis

Statistics is basically descriptive with continuous variables that are described as mean + standard deviation (SD) and categorical variables as relative frequency.

### Ethics and Consent

The present work was approved by the Research Ethics Committee of the Hospital Israelita Albert Einstein (CAAE 34718220.7.1001.0071).

## Results

**Fig. 1:**
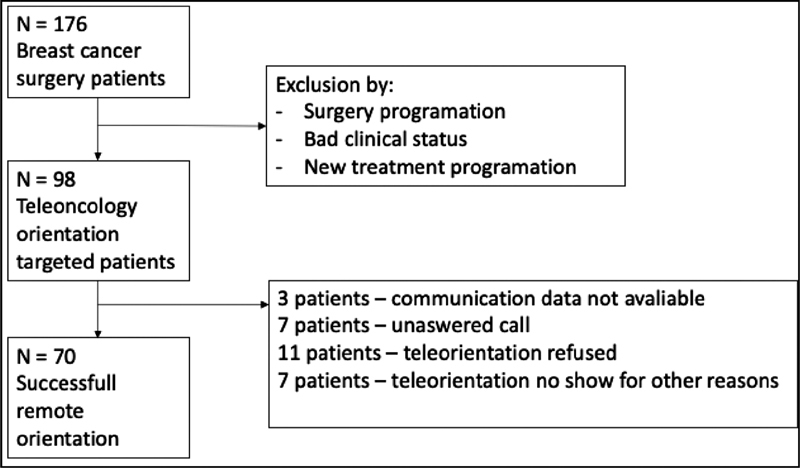
Study flowchart.

A total of 176 eligible patients scheduled for consultation from 04/28/2020 to 07/28/2020 were sequentially evaluated and 98 patients were included. Eleven patients refused to receive the teleorientation, 7 patients did not answer the call, 3 patients had no communication data, and 11 did not show up at the teleorientation for various reasons. Of the remaining 98 patients, 86 (87.7%) had prior scheduled consultations. Seventy patients (71.4%) successfully undertook the teleorientation ( Fig. 1 ).

In 57 (81.5%) cases, the appointments were directly made with the patients, while in 13 (18.5%) cases, the appointments were made with relatives. Of the teleoriented patients, 67 (95.9%) had cell phones and only 42 (60%) had an email address. The presence of a family member was essential to proceed with the evaluation in 16 (22.9%) teleconsultations ( Table 2 ).

**Table 2:** Scheduling characteristics

Variable	Description
	**(** ***n*** ** = 70)**
Responsible scheduling person, *n* (%)	
Patient	57 (81.5%)
Relative	13 (18.5%)
Own cell phone, *n* (%)	67 (95.9%)
Own e-mail adress, *n* (%)	43 (60%)
Need for help from family member	16 (22.9%)

All 70 patients were female, with an average age of 54.9 ± 13 years old. The average home to clinic distance was 15.4 ± 9.2 Km, and the time spent in travel was 33.6 ± 14.3 minutes by car and 72.3 ± 29 minutes by public transportation. Demographically, the number of adults per household was 2.9 ± 0.9, and the number of children per household was 0.5 ± 0.8. The monthly family income was 2.3 (±1.3 times) minimum wages. A Brazilian minimum wage is ∼ 216 US dollars (August 2020). Regarding education, 40 patients provided information and 3 (4.3%) were illiterate, followed by 19 (27.1%) who were literate but had not completed elementary school; 5 (7.1%) had completed elementary school, 11 (15.7%) had not completed middle school, 16 (22.8%) had completed middle school, and 2 (2.8%) had completed high school ( Table 3 ).

**Table 3:** Demographic data

Variable	Description
	( *n* = 70)
Female gender, *n* (%)	70 (100)
Age (years old), mean ± SD	54.9 ± 13
Distance between home and mastology clinic (Km), mean ± SD	15.4 ± 9.2
Time-distance by car between home and mastology clinic (min), mean ± SD	33.6 ± 14.6
Time-distance by public transportation between home and mastology clinic (min), mean ± SD	72.3 ± 29
Adults per household, mean ± SD	2.9 ± 0.9
Children per household, mean ± SD	0.5 ± 0.8
Family income (minimum wage times), mean ± SD	2.3 ± 1.3
Education data	40 (57.1%)
Illiterate	3/40 (4.3%)
Literate without completing elementary school	19/40 (27.1%)
Elementary school	5/40 (7.1%)
Incomplete middle school	11/40 (15.7%)
Middle school	16/40 (22.8%)
High school	2/40 (2.8%)

Abbreviation: SD, Standard deviation.

**Fig. 2:**
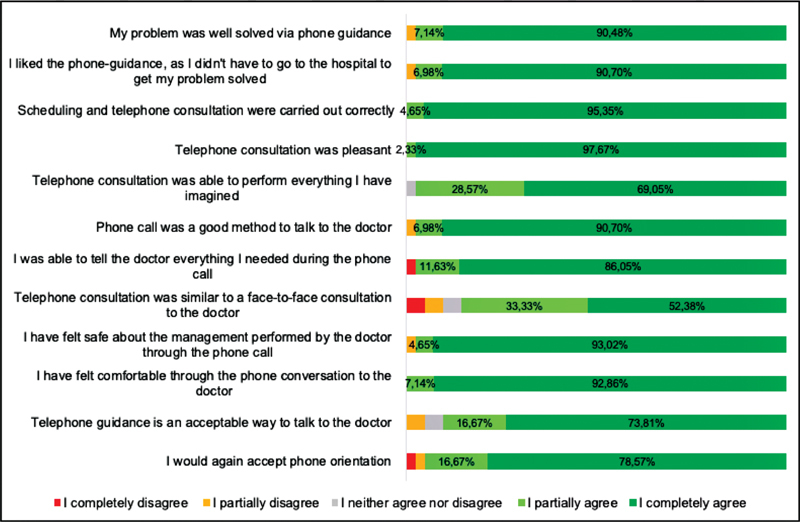
Patient satisfaction during a teleconsultation.

The Likert-based questionnaire was answered by 43 (61.4%) patients. Overall, the teleoncology orientation of the patients was classified as very positive by 41 (95.3%) patients. Specifically, regarding the questionnaire items, 43 (100%) of the patients scored 4 or 5 (agreed that the ease of use was beneficial) concerning the easiness, followed by 42 (97.2%) for the interface quality, 41 (95.3%) for both usefulness and interaction quality, 40 (93%) for satisfaction and interest in future use, and, finally, 39 (90.6%) for reliability ( Fig. 2 ).

## Discussion

The COVID-19 pandemic profoundly changed the regulation of telemedicine services in areas of medicine including cancer care. 10 In the present study, oncologic medical staff faced many challenges raised by the implementation of teleconsultations when the pandemic occurred. The first and complex problem was that of getting the approval of the main management board of the hospital, including a change in scheduling flow, allocation of people and resources, acquisition of new equipment, and the need for recognition and regulation of hours spent on remote care. The implementation of a telemedicine service requires training of local staff and patients for its greater effectiveness. 8 Disagreement over the training strategy at the hospital chosen for the present study delayed the implementation of the strategy. There was a formal teleconsultation appointment to provide psychological and operational preparation (for example, family member present, adequate home environment) for assimilating orientation optimization. This simple workflow probably contributed to the high satisfaction level with the teleconsultations expressed by the patients.

The medical team usually experiences certain restrictions during remote evaluation. 11 In the present study, although the breast surgeons were not experienced in virtual care, all patients had previously been screened, and therefore, face-to-face consultation with them would not have added value to their ongoing care. These situations were: a) communication of benign diagnosis and/or normal test result; b) return during neoadjuvant therapy without any active conduct, and c) request for routine tests. In these situations, the only real difference from the face-to-face assessment would be the absence of the physical examination, which would, anyway, be devoid of clinical relevance in the context of monitoring these patients. 6 12 The profiles of these patients facilitated the beginning experience of the mastologists in teleorientations. Nevertheless, internal unpublished data shows that the impressions of our medical team regarding teleoncology in the same study are slightly worse than the those of our patients, suggesting a cultural barrier to better acceptance of the implemented processes by the providers, and the need to optimize the tools and resources, and leading technical expertise for doctors. 13

Telemedicine has already been shown to be feasible among low-income populations, as well as for cancer patients. 14 15 The present study exposes the challenge of remote care for people with multiple clinical and social vulnerabilities. Breast cancer patients are at high risk of severe presentation of COVID-19 and for the risk associated with the interruption in treatment, as well as a negative psychological impact. 16 Maintaining service to this population while observing social distancing was, apparently, a reason for high satisfaction, along with perceived usefulness, reliability, and interest in the future remote evaluation.

Brazilian laws ensure the participation of citizens in the management of health services. 17 However, patient satisfaction has only recently begun to emerge in our country. At this point, the present work brings to light and discussion the issue of user satisfaction that can be used as a parameter that allows the evaluation of the communication pattern and assists in decision-making regarding the adoption of a specific tool. 18 Individual characteristics such as age (older patients), educational level (less formal education), and social class (higher social classes) can, apparently, positively influence satisfaction with health services. 19 Our patients had a blend of the following characteristics: younger patients, of low social class, and low educational level.

The great distances and the long mean travel time for face-to-face consultation are markers of this population served by the Brazilian Public Health Care system (SUS, in the Portuguese acronym). On the other hand, they constitute a population with great potential to benefit from the application of telemedicine due to cost-saving. 13 15 Difficulties in providing teleorientation were foreseen as a very low-income population was selected for it. The subjects had restricted access to current basic technology (such as the internet and computers) and a low level of education. However, in their case, the simultaneous presence of family members during the consultation and the efforts of the medical team ensured effective care, good interface quality in most cases, and final patient perception of great feasibility of the remote evaluation. It is interesting to point out that the adoption of more robust technologies, such as the use of video and sound tools and the use of digital scheduling of appointments, physical examination, and electronic prescriptions could potentially further increase the effectiveness of telemedicine in our scenario.

Teleoncological orientation of the selected patients was feasible, and they found it highly satisfactory. Just as important was the possibility of navigating the patient with breast cancer through the institutional care protocol. In the resource scarcity context, navigation is often neglected; public health care patients are frequently at risk of missing optimal follow-up by missing physical examinations and appointments. The integrated electronic medical record was fundamental to the success of the program because it allowed weekly remote medical meetings with an in-depth discussion of disease management, with better security and effectiveness, and could have extended access to better patient understanding and greater adherence to the treatment.

As a limitation of the present study, a relatively small number of patients included ( *n* = 70) and who answered the questionnaire ( *n* = 43) was available. Studies with a larger number of patients are necessary to observe, with a higher level of evidence, patient satisfaction with this type of approach. Unfortunately, since the answered questionnaire was anonymous, it was not possible to perform a specific clinical and sociodemographic analysis of the group of responding patients. Even so, there was a good acceptance of the teleconsultation by the included participants.

Although the benefits of teleoncology for remote populations are evident, its benefits for populations that suffer greatly from lack of resources have been evaluated but little in the past. 20 The possible benefits of telemedicine are enormous in countries with high social heterogeneity, such as Brazil. 21 Although the trigger for the implementation of telemedicine in this oncology service was the COVID-19 pandemic, its expansion is important for the better perception of the health system by the patient, the rational use of individual and public resources, and for maintaining the role of the doctor-patient relationship as central to cancer care.

## Conclusion

Teleoncology orientation of low-income breast cancer patients is most feasible and leads to high patient satisfaction. Expansion and adoption of the telemedicine program are essential even after the pandemic has passed, and its implementation demands investment, regulation, and training of medical staff and patients.
